# Estivation-responsive microRNAs in a hypometabolic terrestrial snail

**DOI:** 10.7717/peerj.6515

**Published:** 2019-02-20

**Authors:** Myriam P. Hoyeck, Hanane Hadj-Moussa, Kenneth B. Storey

**Affiliations:** Institute of Biochemistry, Departments of Biology and Chemistry, Carleton University, Ottawa, Ontario, Canada

**Keywords:** *Otala lactea*, miRNA, Mollusc, Metabolic rate depression

## Abstract

When faced with extreme environmental conditions, the milk snail (*Otala lactea*) enters a state of dormancy known as estivation. This is characterized by a strong reduction in metabolic rate to <30% of normal resting rate that is facilitated by various behavioural, physiological, and molecular mechanisms. Herein, we investigated the regulation of microRNA in the induction of estivation. Changes in the expression levels of 75 highly conserved microRNAs were analysed in snail foot muscle, of which 26 were significantly upregulated during estivation compared with controls. These estivation-responsive microRNAs were linked to cell functions that are crucial for long-term survival in a hypometabolic state including anti-apoptosis, cell-cycle arrest, and maintenance of muscle functionality. Several of the microRNA responses by snail foot muscle also characterize hypometabolism in other species and support the existence of a conserved suite of miRNA responses that regulate environmental stress responsive metabolic rate depression across phylogeny.

## Introduction

Native to seasonally arid regions of southern Europe, the milk snail, *Otala lactea*, experiences seasonal exposures to high temperatures and limited food and water. To withstand these harsh environmental conditions, the snail spends months in a dormant state known as estivation (Ramnanan & Storey, 2006a; Ramnanan & Storey, 2006b). Estivation is a survival strategy characterized by a strong reduction of basal metabolic rates to <30% of normal resting rates. Such metabolic rate depression (MRD) is a widespread survival strategy used by both vertebrates and invertebrates as a means of reprioritizing energy usage towards vital cellular processes, and preserving/stabilizing macromolecules to maximize survival when faced with unfavourable environmental conditions (Storey, 2002; Storey & Storey, 2004; Storey & Storey, 2010). However, retreating into an estivating state exposes the snail to cellular stresses including dehydration, hypoxia (oxygen limitation), hypercapnia (elevated CO_2_ levels), and acidosis (Churchill & Storey, 1989; Storey, 2002; Storey & Storey, 2012). As such, the milk snail has developed various adaptations to overcome estivation-associated stresses and to facilitate survival over prolonged periods of dormancy (Storey, 2002; Storey & Storey, 2012).

Although dormancy is a life-saving strategy for many animals, this phenomenon is far from being a simple feat. Indeed, animals need to undergo profound biochemical changes (alterations to gene and protein expression and enzyme regulation) that modulate cellular metabolism to allow for safe transitions into dormancy while stabilizing long-term viability. As such, these regulatory mechanisms must be rapid, reversible, easily inducible, and have a low energy demand (Storey & Storey, 2007). MicroRNAs (miRNAs) possess all of these characteristics, making them excellent candidate regulators of hypometabolism (Biggar & Storey, 2011). Indeed, miRNAs have been shown to be master regulators of virtually all cellular processes, exerting widespread control over activities including signal transduction pathways, cell cycle, apoptosis, atrophy, energy metabolism, and many others. These small non-coding RNA molecules act post-transcriptionally to modulate gene expression by permanently repressing translation by directing mRNA transcripts to degradation, or temporarily targeting mRNAs into storage in stress granules or P-bodies (Nilsen, 2007). In addition, miRNAs provide remarkable regulatory potential and flexibility since an individual miRNA type can target multiple different mRNA transcripts, and equally, a single type of mRNA transcript can be targeted by diverse miRNAs (Mazière & Enright, 2007; Biggar & Storey, 2011).

MiRNA-mediated regulation of gene expression has been well established as part of a typical stress response (e.g., during ischemic injury, cardiac hypertrophy, etc.) and recent studies have highlighted their role in multiple forms of environmental stress-induced hypometabolism (Biggar & Storey, 2018) including mammalian hibernation (Hadj-Moussa et al., 2016), hypoxia/anoxia tolerance (Biggar et al., 2012; Hadj-Moussa et al., 2018), estivation (Chen et al., 2013; Wu, Biggar & Storey, 2013; Chen & Storey, 2014; Luu & Storey, 2015), insect cold-hardiness/diapause (Lyons et al., 2015), and freeze tolerance (Biggar et al., 2012; Lyons et al., 2013; Hadj-Moussa & Storey, 2018). These studies linked differential miRNA expression to protective actions in the hypometabolic state including minimizing apoptosis and muscle wasting, limiting non-essential energy-expensive processes, promoting cell cycle arrest, and differentially regulating selected signalling cascades (Biggar & Storey, 2011). Additionally, miRNAs have also been shown to mediate survival responses to desiccation and osmotic and heat stress in marine molluscs (Zhou et al., 2014; Zhao et al., 2016; Chen et al., 2017). Consequently, we predicted that miRNAs are also likely to be involved in facilitating the successful transition into estivation in *O. lactea*. In this study, we examined the expression levels of 75 conserved miRNAs in snail foot muscle, comparing active snails to those in prolonged estivation, to better understand the molecular processes involved in mediating estivation and stress survival. This represents the first report of differential miRNA expression in an estivating land snail.

## Materials and Methods

### Animal care and treatment

*Otala lactea* snails, imported from Morocco, were purchased from a local seafood retailer. In the laboratory, snails were treated as described by Ramnanan, Groom & Storey (2007). All animals were held at ∼22 °C in plastic boxes lined with damp paper towels and fed shredded carrots and cabbage (sprinkled with crushed chalk) every 2–3 days. After 4 weeks, half of the snails were moved to a container with dry paper towels and no food where they quickly entered estivation. Other snails were maintained under active conditions. After 10 days, active (control) and estivating snails were euthanized, and foot muscle was dissected out, flash frozen in liquid nitrogen, and stored at −80 °C.

### Total RNA isolation

Total RNA was isolated from foot muscle as previously described by Hadj-Moussa et al. (2018). In brief, samples were homogenized in Trizol (Invitrogen), RNA was isolated using chloroform, and precipitated with isopropanol. RNA pellets were resuspended in RNase-free water, and samples were stored at −20 °C until use.

### Polyadenylation and stem-loop reverse transcription

RNA samples were prepared for miRNA analysis as described by Biggar, Wu & Storey (2014). Briefly, RNA samples were polyadenylated using a PolyA tailing kit (Cat #.PAP5104H; Epicenter, Madison, WI, USA). Reverse transcription was performed to generate cDNA from polyadenylated RNA using a stem-loop adapter primer (Table S1). Serial dilutions of the cDNA were prepared and stored at −20 °C until use.

### Primer design

Snail miRNA-specific forward primers were designed using the annotated gastropod (*Lottia gigantea*) miRNA sequences and synthesized by Integrated DNA Technologies (Biggar, Wu & Storey, 2014). The primers were validated by assessing the miRNA sequence conservation of *L. gigantea* against other molluscs including gastropods (*Aplysia californica*, *Lymnaea stagnalis*), a bivalve (*Crassostrea gigas*) and a cephalopod (*Octopus bimaculoides*) using NCBI BLASTn and CLUSTAL OMEGA. All miRNA-specific forward primers were designed with a modified short adapter at the 5′ end of the mature miRNA sequence, with the following general sequence 5′-ACA CTC CAG CTG GGN NNN NNN NNN NNN NN-3′, where N denotes miRNA-specific sequence binding regions (Biggar, Wu & Storey, 2014) (Table S1).

### Relative miRNA quantification

qRT-PCR reactions were preformed as previously described (Pellissier et al., 2006) following MIQE guidelines (Bustin et al., 2009) using a BioRad MyIQ_2_ Detection System. Target-specific forward primers were used in conjunction with a universal reverse primer to amplify each miRNA target (Table S1). A post-run melt-curve was performed after all PCR reactions to ensure the amplification of a single product. Reactions that amplified multiple products were rejected.

Relative miRNA expression levels were calculated using the comparative ΔΔCq method as previously described (Luu & Storey, 2015). In brief, raw Cq values were converted to the 2^−Cq^ form and normalized to the 5S rRNA reference gene. The 5S rRNA gene displayed abundant and stable expression in *O. lactea* foot muscle under control and estivation conditions making it a suitable reference gene (Schmittgen & Livak, 2008). Standardized values were expressed as mean relative expression (mean ± SEM, with *n* = 3 − 4 independent biological replicates). Statistical analysis used a two sample Student’s *t*-test (Table S2).

## Results and Discussion

The roles of transcriptional, translational, and post-translational modifications in supporting MRD have been extensively analyzed in multiple animal systems over the years (including in hibernation, daily torpor, anaerobiosis, and estivation), whereas post-transcriptional miRNA-mediated metabolic regulation has only recently become evident (Storey & Storey, 2004; Biggar & Storey, 2011; Storey, 2015). Indeed, various post-translational regulatory controls on *O. lactea* signal transduction and metabolic enzymes have been reported (Brooks & Storey, 1990; MacLean et al., 2016; Ramnanan et al., 2009; Ramnanan et al., 2010; Ramnanan & Storey, 2006a; Ramnanan & Storey, 2006b; Whitwam & Storey, 1990) but, to date, the role of miRNA had not been explored. This study evaluates this level of post-transcriptional control. Studies have shown that *O. lactea* can arouse from dormancy in as little as 5–10 min and be immediately capable of extending its foot from the shell (Storey, 2002). As such, the readily inducible, rapid, and reversible nature of miRNAs makes them excellent candidates for quickly mediating metabolic changes needed to support entry into or exit from dormancy in the snail. Although the genome of *O. lactea* has not been sequenced, the high degree of evolutionary conservation of miRNAs across species, including both vertebrates and invertebrates, allowed for this miRNA analysis (Friedman et al., 2009).

**Figure 1:**
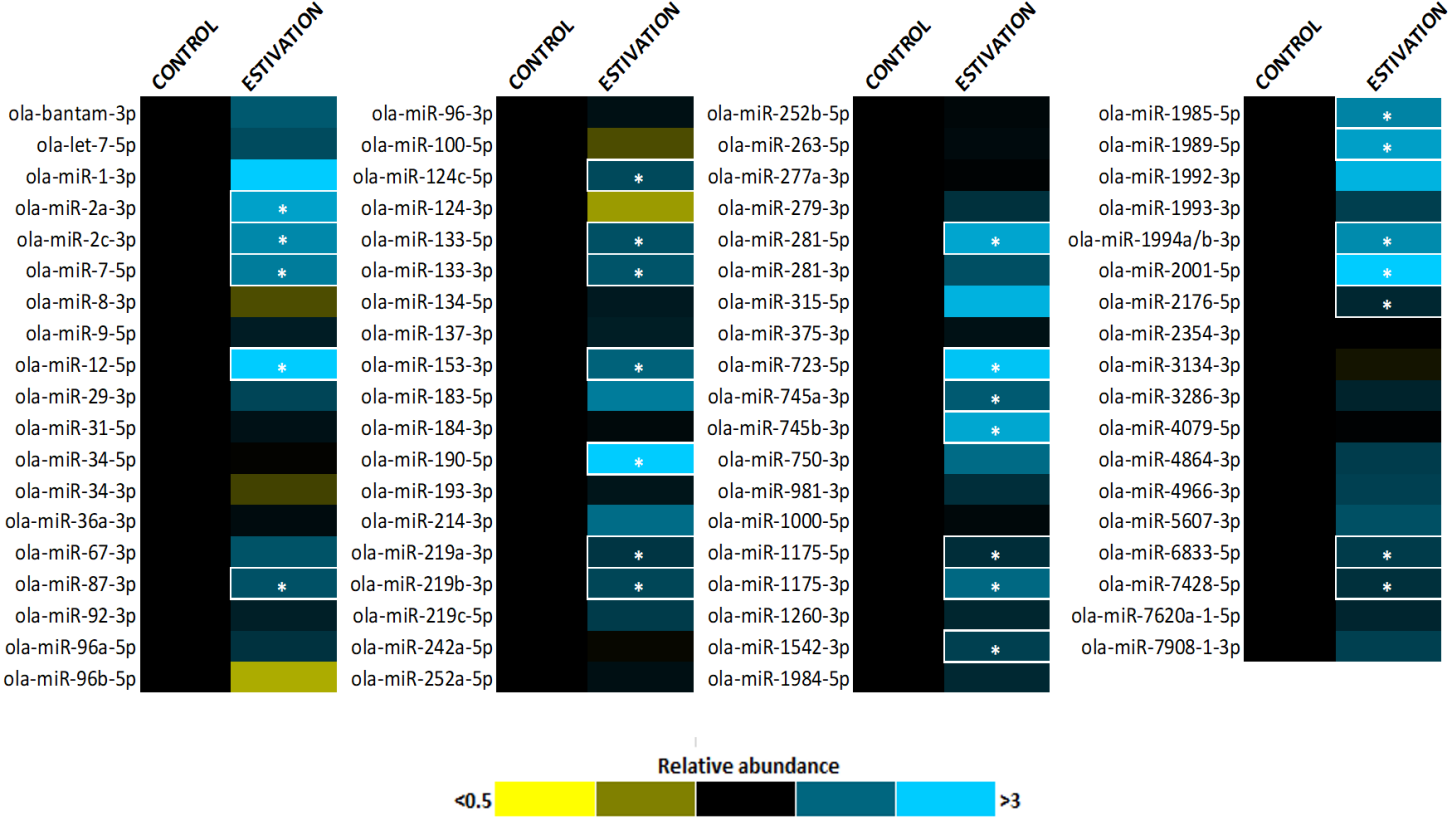
Heatmap of miRNA expression during estivation. Heatmap displaying estivation-induced changes in miRNA abundance in the foot muscle of *O. lactea*, relative to controls, as determined by RT-qPCR. MiRNA abundance was standardized against the 5S rRNA reference gene. Data are means ± SEM, *n* = 3 − 4 biological replicates. Statistical analysis was performed using a Student *t*-test and expression levels significantly different from control (*p* < 0.05) are indicated by *.

**Figure 2:**
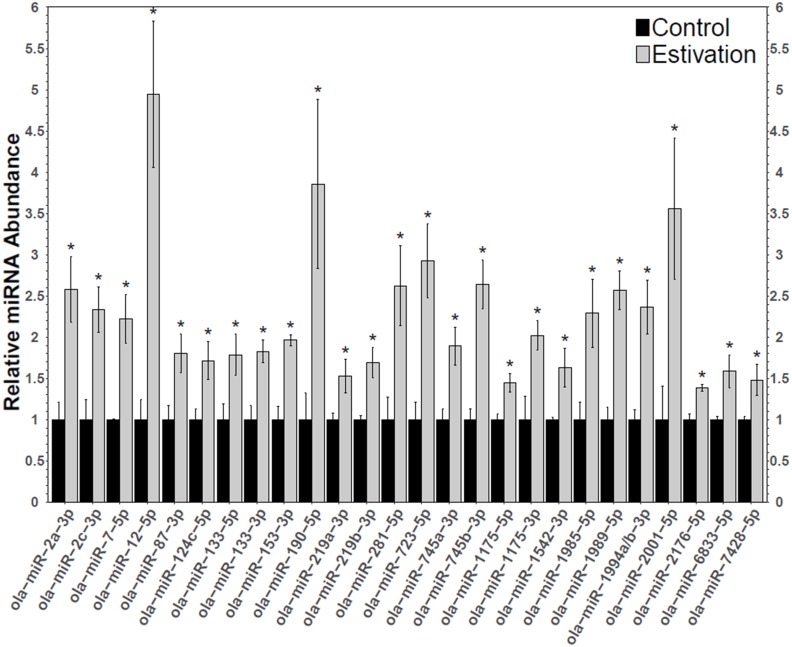
Differentially expressed miRNAs during estivation. Changes in the relative abundance of 26 miRNAs in the foot muscle of estivating snails, *O. lactea*, as determined by RT-qPCR. MiRNA abundance was standardized against the 5S rRNA reference gene. Data are means ± SEM, *n* = 3 − 4 biological replicates. Statistical analysis was performed using a Student *t*-test and expression levels significantly different from control (*p* < 0.05) are indicated by *.

A total of 75 miRNAs were successfully detected (Fig. 1), of which 26 were significantly upregulated, the increase during estivation ranging from 1.39–4.95 fold relative to expression in control foot muscle (Fig. 2; Table S2). Only five of the assessed miRNAs were downregulated but none of these showed a significant change compared with controls. Among the subset of upregulated miRNAs, miRNAs *ola-miR-2a-3p, ola-miR-12-5p*, *ola-miR-190-5p, ola-miR-281-5p, ola-miR-723-5p, ola-miR-745b-3p*, *ola-miR-1989-5p, ola-miR-2001-5p* showed strong upregulation by over 2.5 fold. The 26 miRNAs significantly elevated in the estivating state, suggest that their target genes were translationally suppressed during dormancy. Overall, these significantly upregulated miRNAs were implicated in regulating cell survival mechanisms that constituted three main functional groups: (1) anti-apoptosis, (2) tumour suppression, and (3) muscle maintenance responses. The present study showed elevated levels of five anti-apoptosis miRNAs (miR-2a-3p, miR-2c-3p, miRNA-124c, miRNA-153, and miRNA-190) in foot muscle (Gennarino et al., 2012; Wu et al., 2013; Dwivedi, 2011; Jia et al., 2016). The miR-2 family is an invertebrate-specific group of miRNAs involved in neural development and maintenance (Marco, Hooks & Griffiths-Jones, 2012). However, they have also been shown to specifically target the pro-apoptotic genes *reaper*, *grim*, and *sickle*, suggesting that miR-2 prevents apoptosis under stress and contributes to long-term life extension (Gennarino et al., 2012). Indeed, this miRNA was also upregulated in the intertidal snail *Littorina littorea* under freezing and anoxia stresses, as well as in the Humboldt squid *Dosidicus gigas* in response to hypoxia (Biggar et al., 2012; Hadj-Moussa et al., 2018). This suggests that miR-2 upregulation is a conserved invertebrate response to cellular stresses imposed by harsh environmental conditions.

Another anti-apoptotic miRNA identified was miR-153, a miRNA that is known to target the PTEN protein phosphatase to relieve Akt suppression (Wu et al., 2013). Akt signal transduction regulates central cell functions including metabolism, cell growth, transcriptional regulation, and cell survival (Zhang et al., 2011). Akt directly regulates apoptosis and promotes survival by targeting pro-apoptotic Bcl-2 related proteins, Forkhead factors (FOX), CREB, and p53 activity (Song, Ouyang & Bao, 2005; Zhang et al., 2011). As such, Akt has a key regulatory function to suppress apoptosis during dormancy. Indeed, a previous study of *O. lactea* reported elevated levels of Akt that in turn led to reduced levels of BAD and FOXO which acted to promote anti-apoptotic processes during estivation (Ramnanan, Groom & Storey, 2007). The role of miR-153 in stress survival has also been documented in the sea cucumber in response to hypoxia, indicating that miR-153 may also exert conserved functions in invertebrates (Zhao et al., 2014). Furthermore, miR-124 is known to relieve the suppression of CREB, a transcription factor responsible for mediating Akt-induced expression of anti-apoptotic factors (i.e., Bcl-2 and Mcl-1) (Du & Montminy, 1998; Song, Ouyang & Bao, 2005). As such, the upregulation of miR-124 in *O. lactea* may further promote survival by enhancing anti-apoptotic responses. Members of the miR-124 family were also upregulated in hibernating bats and thirteen-lined ground squirrels, suggesting a conserved function of these miRNAs in facilitating hypometabolism (Yuan et al., 2015; Wu et al., 2016). Lastly, although the exact function of miR-190, another miRNA targeting Akt members, is not fully understood, it has been shown to suppress TGFβ and FOXP2, two pro-apoptotic factors (Myatt & Lam, 2007; Ramesh, Wildey & Howe, 2011; Gennarino et al., 2012; Jia et al., 2016). Dormancy is associated with multiple potential intracellular stresses (e.g., oxidative stress, accumulation of waste products, protein damage, etc.) that can lead to metabolic injury and even cell death (Storey & Storey, 2007). As such, stress-tolerant animals rely on anti-apoptotic processes to prolong survival, as seen in freeze-tolerant wood frogs, and hibernating ground squirrels and bats (Chen et al., 2008; Rouble et al., 2013; Gerber, Wijenayake & Storey, 2016). Results from the present study suggest that the milk snail induces the expression of anti-apoptotic miRNAs during estivation to prolong survival.

One of the most energy-expensive activities of an organism is the proliferation of cells to support growth and development, or replacement of damaged cells (Mazia, 1961; Storey & Storey, 2004). As such, cell cycle arrest is a central energy-conserving strategy employed by most species entering a hypometabolic state including freeze-tolerant frogs, hibernating squirrels, and anoxia-tolerant turtles (Biggar & Storey, 2012; Wu & Storey, 2012; Zhang & Storey, 2012). Herein, two tumour-suppressing miRNAs, miR-7 and miR-219, were upregulated in *O. lactea* foot muscle during estivation. MiR-7 is an evolutionarily conserved miRNA that supresses cell growth and proliferation via numerous mechanisms such as targeting Akt and Erk1/2 (Kalinowski et al., 2014). Likewise, miR-219 inhibits Erk1/2 signaling by potentially targeting the receptor tyrosine kinases EBB3 (epidermal growth factor receptor family), although this finding requires further analysis (Lei et al., 2013). The Erk signal transduction cascade regulates various growth and proliferation cell processes including cell cycle progression, cell survival, differentiation, metabolism, transcription, amongst others (Roskoski, 2012). As such, the upregulation of miR-7 and miR-219 during estivation suggests that *O. lactea* enhances long-term survival via energy reprioritization by suppressing cell cycle progression and cell proliferation.

Despite long periods of inactivity during dormancy, the milk snail displays no signs of muscle disuse atrophy and has been shown to regain muscle activity within minutes of arousal (Storey, 2002). The snail’s reliance on its foot muscle to attach itself to its surroundings suggests that it has adaptive strategies in place to overcome the onset of foot muscle atrophy that would be detrimental to survival. In the present study, miR-133 was upregulated during estivation. MiR-133 is a muscle-specific miRNA that has been shown to regulate myosin genes (i.e., MEF and MyoD) and muscle signaling pathways (i.e., MAPK and Erk1/2), thereby protecting against atrophy and promoting muscle differentiation (Biggar & Storey, 2011; Feng et al., 2013). Both MEF2 and MyoD are well-characterized muscle transcription factors that regulate muscle plasticity, differentiation, proliferation, cell survival, and apoptosis (Tessier & Storey, 2016), and together these factors have been implicated in stress survival in numerous hypometabolic species (Black & Olson, 1998; Hadj-Moussa et al., 2016; Tessier & Storey, 2016; Hoyeck, Hadj-Moussa & Storey, 2017). As such, the estivation-induced upregulation of miR-133 alludes to its role in preserving muscle function, suppressing cell cycle progression, and promoting survival in *O. lactea*. These findings are supported by previous discoveries of miR-133 upregulation in response to hypoxia in the jumbo squid and following freezing and anoxia in *L. littorea*, suggesting that miR-133 may provide a conserved mechanism for maintaining muscle function during hypometabolism (Biggar et al., 2012; Hadj-Moussa et al., 2018).

Our knowledge of miRNA functions in non-model species is far from complete. Consequently, the function of several miRNAs that showed upregulation in this study remains unknown. To our knowledge, this is the first report suggesting that miR-87, -281, -723, -745, -1542, -1989, -1994, -2176, -6833 and -7428 may be implicated in stress survival. Only one report on osteoarthritis suggests a role for miR-6833 in cell proliferation, metabolism, homeostasis, and response stimuli (Balaskas et al., 2017). Likewise, the function of miR-2001 has not been elucidated, although it has been associated with the stress response in marine lifeforms, including the squid *D. gigas* (Zhou et al., 2015; Hadj-Moussa et al., 2018). Lastly, very limited information is available on miR-12, -1175, or -1985, with only one reported finding of differential expression of these miRNAs in response to hypoxia in *D. gigas*, suggesting a potential role in invertebrate (or possibly only mollusc) hypometabolic regulation (Hadj-Moussa et al., 2018).

## Conclusion

This study is the first to explore the stress-induced regulation of miRNAs in an estivating land snail. All estivation-responsive miRNAs identified in this study were upregulated, suggesting a role for miRNAs in energy reprioritization for vital processes by reducing mRNA translation in the hypometabolic state. Our findings indicated that estivation-induced miRNAs are implicated in anti-apoptotic, tumour suppressive, and muscle maintenance responses. Many of our findings follow similar expression patterns reported in other hypometabolic models, suggesting that miRNAs serve a conserved role necessary for survival during prolonged dormancy. Our present study suggests that miRNAs regulate these survival mechanisms by targeting the Akt and Erk1/2 signaling pathways, as well as myosin genes. Overall, this study deepens our understating of the molecular mechanisms involved in transitioning to and from a hypometabolic state and highlights conserved miRNA-meditated invertebrate and stress-responsive adaptations.

##  Supplemental Information

10.7717/peerj.6515/supp-1 Table S1 MicroRNA primers Primers used for miRNA analysis of *O. lactea* foot muscle including miRNA-specific forward primers, universal reverse primer, reference gene primers, and stem-loop adapter used for reverse-transcription.

10.7717/peerj.6515/supp-2 Table S2 Relative miRNA expression Relative abundance of 75 miRNAs in the foot muscle of *O. lactea* during estivation, relative to controls. MiRNA abundance was evaluated by RT-qPCR and standardized against the 5S rRNA reference gene. Data are means ± SEM, *n* = 3 − 4 biological replicates. Statistical analysis was performed using a Student *t*-test where expression levels significantly different from control (*p* < 0.05) are indicated by *.

10.7717/peerj.6515/supp-3 Table S3 Estivating snail miRNA (raw data)

## Additional Information and Declarations

### Data Availability

The following information was supplied regarding data availability: The raw data are available in a Supplemental File.
